# Canadian permafrost stores large pools of ammonium and optically distinct dissolved organic matter

**DOI:** 10.1038/s41467-020-18331-w

**Published:** 2020-09-09

**Authors:** J. Fouché, C. T. Christiansen, M. J. Lafrenière, P. Grogan, S. F. Lamoureux

**Affiliations:** 1 LISAH, Univ Montpellier, INRAE, IRD, Institut Agro, 34060 Montpellier, France; 2 Department of Geography and Planning, Queen’s University, Kingston, ON K7L 3N6 Canada; 3 Center for Permafrost (CENPERM), Department of Geoscience and Natural Resource Management, University of Copenhagen, DK-1350 Copenhagen, Denmark; 4 Department of Biology, Queen’s University, Kingston, ON K7L 3N6 Canada

**Keywords:** Carbon cycle, Cryospheric science

## Abstract

Permafrost degradation may lead to mobilization of carbon and nutrients and enhance microbial processing rates of previously frozen organic matter. Although the pool size and chemical composition of dissolved organic matter (DOM) are fundamental determinants of the carbon cycle in Arctic watersheds, its source within the seasonally thawing active layer and the underlying permafrost remains largely uncharacterized. Here, we used 25 soil cores that extended down into the permafrost from nine sites across Arctic Canada to quantify dissolved organic carbon (DOC) and nitrogen stocks, and to characterize DOM optical properties. Organic permafrost stores 5–7 times more DOC and ammonium than the active layer and mineral permafrost. Furthermore, the permafrost layers contain substantial low molecular weight DOM with low aromaticity suggesting high biodegradability. We conclude that soil organic matter stoichiometry and cryogenic processes determine permafrost DOM chemistry, and that thawing will mobilize large amounts of labile DOC and ammonium into Arctic watersheds.

## Abstract (web summary)

Arctic warming leads to permafrost thaw and release of previously frozen organic matter and nutrients, but characterization of these pools is lacking. Here the authors use soil cores across Northern Canada to investigate dissolved organic matter and nitrogen stored in permafrost soils.

## Introduction

Global warming will lead to massive declines in the extent of northern permafrost soils during this century^1^. Permafrost degradation occurs through either a gradual thickening of the seasonally thawed active layer or the development of thermokarst landforms as land surface subsidence follows thawing of ice-rich permafrost^2^. Permafrost-affected soils store an estimated 1000 ± 150 Pg organic carbon at 0–3 m depth^3^ and climate warming-induced thaw therefore has the potential to increase C emissions to the atmosphere through enhanced microbial decomposition of organic matter^2^. In addition to being a reactive and easily mobilized pool of soil organic matter (SOM), dissolved organic matter (DOM) is an important substrate for microorganisms and a crucial component of overall net ecosystem carbon balance in Arctic watersheds^4^. Yet, research on the permafrost DOM pool size and composition remains scarce^5–8^ despite a clear need to evaluate permafrost thaw as a potential positive feedback on climate change. This study is the first to characterize the permafrost DOM pool systematically and to estimate the dissolved nitrogen pool across Northern Canada, a severely underrepresented region in climate-change research^9^.

Permafrost degradation alters ground surface hydrology and strongly modifies the amount and composition of DOM and nutrients which are mobilized from terrestrial to aquatic ecosystems^10^. Warming and permafrost thaw increase dissolved inorganic nitrogen (DIN) pool sizes through organic matter mineralization and additional permafrost DIN release^11^, promoting deep-rooted plant growth^12^, changing microbial and plant community composition^13^ while increasing microbial metabolic efficiency^14^, and significantly affecting carbon and nitrogen cycling in northern ecosystems^15^.

In addition to the amount of DOM exported, watershed carbon emissions partly depend on DOM biodegradability, which is determined by its chemical composition^16^, its sorption to soil minerals^17^, stage of degradation and photo-oxidation^18^, abiotic conditions^19^, nutrient availability^18^, and microbial community functionality^16^. Current evidence suggests that a substantial portion of permafrost DOM is relatively quickly mineralized by microbes and/or photo-oxidized after permafrost thaw during its transport from soils to rivers and towards the Arctic Ocean^5^. However, to date, the characterization of the composition and lability of permafrost DOM remains limited to only a few Arctic locations^5,6,10,19^.

In order to characterize and quantify DOM and DIN pools stored in Canadian permafrost soils, we utilized 25 active layer and permafrost (upper 3 m) cores from a wide range of permafrost-affected settings that varied in formation histories, ages, surficial geology, and climate conditions. For all cores, we grouped water extracts from the active layer or permafrost samples into either organic (soil C ≥ 12%) or mineral (soil C < 12%) layers. Soil samples were gently leached during a short period by using milliQ water in order to simulate the leaching that would occur immediately upon permafrost thaw. We analyzed each sample for dissolved organic carbon (DOC), total dissolved nitrogen (TDN), and DIN, and DOM optical properties using absorbance and fluorescence spectroscopy. In addition to using optical indices to characterize DOM aromaticity and molecular weight^20^, we performed PARAFAC modeling to identify the main chemical components explaining the fluorescent DOM pool^21^. By quantifying and characterizing DOM in permafrost soils from a wide range of formation histories, ages, surficial geology, and climate conditions, we are able to highlight the distinct signature of DOM in permafrost layers compare to the active layer and to investigate the controls of DOM character. In addition, we estimate that large of pools of DOM and dissolved nitrogen have the potential to be released in surface waters with active layer thickening or the thaw induced exposure of permafrost.

## Results and discussion

### Properties of permafrost soils

All sites contained syngenetic permafrost in which the active layer and the uppermost permafrost have experienced numerous freeze-thaw cycles since formation during the Holocene^22^. Permafrost organic matter radiocarbon ages ranged from 7850 ± 30 to 830 ± 20 y B.P. (Supplementary Data), with the western sites containing the oldest SOM and the northern Hudson Bay peatlands containing the youngest SOM.

For organic layers, permafrost soil C:N atomic ratios (14.50 [12.61–19.67], median [25^th^–75^th^]) were lower and H:C atomic ratios (0.13 [0.13–0.14]) were greater relative to the active layer, 23.89 [19.33–29.50] and 0.14 [0.12–0.15], respectively (Supplementary Fig. 1). Similarly for mineral layers, permafrost C:N ratios were lower (12.0 [3.23–18.09]) and H:C ratios higher (0.24 [0.15–0.86]) compared to the active layer, 16.21 [13.67–18.87] and 0.17 [0.15–0.21], respectively. For both thermal layers, in organic layers C:N ratios were higher and H:C ratios were lower than in mineral layers.

These stoichiometric properties are typical of boreal and tundra soils (Supplementary Fig. 1)^23^. The higher C:N and very low H:C ratios of the organic layers relative to mineral layers suggest higher contents of condensed aromatic structures originating from peat^24^. Permafrost layers displayed lower C:N properties suggesting different SOM composition (e.g., lignin, tannins, lipids, sugars or amino acids) and an enrichment in microbial biomass relative to the active layer^24^. The absence of a downward trend of C:N and H:C within the permafrost (Supplementary Fig. 1), except at Daring Lake, indicates that soil development and microbial processing were effectively halted soon after permafrost aggradation^23^.

### Active layer and permafrost yields of DOC and nitrogen

**Fig. 1:**
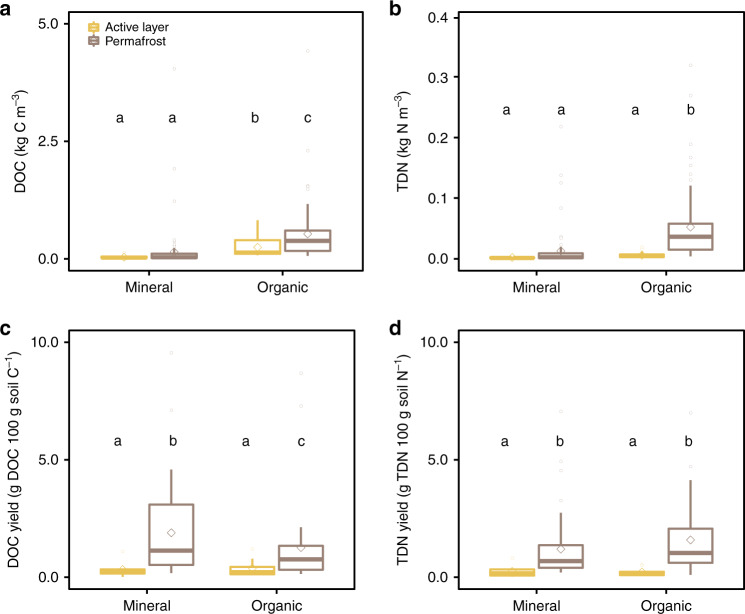
Dissolved organic carbon and total dissolved nitrogen pools and yields in the active layer and permafrost. Samples are grouped by layer type with the following classification: organic layers (soil C ≥ 12%) and mineral layers (soil C < 12%). Letters indicate significant differences between thermal layers and soil types indicated by Mann-Whitney U test. The box plots summarize the distribution of **a** the amounts of dissolved organic carbon (DOC, kg C m^−3^) and **b** total dissolved nitrogen (TDN, kg N m^−3^) and the **c** water extractable yields in DOC (g DOC 100 g soil C^−1^) and **d** TDN (g TDN 100 g soil N^−1^) for each layer type and in the active layer and underlying permafrost (calculated from multiple 25 cm increments). In each box plot, the diamond represents the mean, the horizontal line represents the median, the end of the box the 25^th^ and 75^th^ percentiles, and the lines extending from the box are 1.5 interquartile ranges from the median. Data points outside of the 1.5 interquartile ranges are represented as dots.

DOC content correlated with soil C content in both active layer (*r*^2^ [log–log] = 0.748, *P* < 0.001) and permafrost (*r*^2^ [log–log] = 0.834, *P* < 0.001, Supplementary Fig. 2). DOC and TDN pools were larger in organic layers relative to mineral soil layers (Fig. 1a, b). Organic permafrost yielded greater amounts of DOC and TDN than the active layer and mineral permafrost (Fig. 1a, b). We estimate that organic permafrost layers contained 0.396 [0.178–0.644] kg DOC m^−3^, and 37.6 [15.7–59.8] 10^−3^ kg TDN m^−3^, potentially releasing 5.3 times more DOC and 7.2 times more TDN upon thaw than either of the organic active layer, the mineral active layer, and the mineral permafrost layer (DOC: *Kruskal–Wallis chi-squared* = 103.22, *P* < 0.001, TDN: *Kruskal–Wallis chi-squared* = 85.3, *P* < 0.001, Fig. 1a, b), respectively. DOC pools in both active layer and permafrost were similar to values reported for mineral layers in Pleistocene Yedoma permafrost^5^ and in Orthels and Turbels from interior Alaska^6^, as well as for organic layers in Histels from Alaska and Siberia^6,25^.

Within the active layer, organic and mineral soil samples displayed similar DOC (*W* = 48, *P* = 0.941) and TDN yields (*W* = 35, *P* = 0.963). Both DOC and TDN yields increased in the permafrost relative to the active layer (Fig. 1c, d), with mineral layers displaying greater DOC yields than organic layers in the permafrost (*W* = 1077, *P* = 0.019), extracting 1.3% [0.6–3.1] of soil C and 0.9% [0.3–1.4] of soil N, respectively. Our yields were in the range of values reported for permafrost soils in North America^25,26^.

**Fig. 2:**
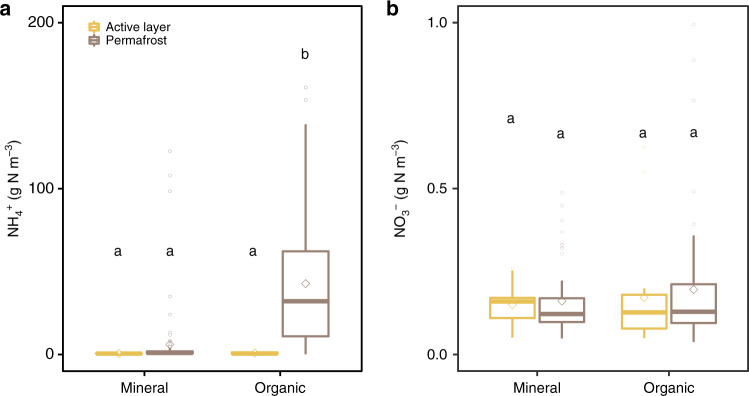
Pools of ammonium and nitrate in the active layer and permafrost. Samples are grouped by layer type with the following classification: organic layers (soil C ≥ 12%) and mineral layers (soil C < 12%). Letters indicate significant differences between thermal layers and soil types indicated by Mann–Whitney U test. The box plots summarize the distribution of **a** the amounts of ammonium (NH_4_^+^) (kg N m^−3^) and **b** nitrate (NO_3_^−^) (g N m^−3^) for each layer nature and in the active layer and permafrost. In each box plot, the diamond represents the mean, the horizontal line represents the median, the end of the box the 25^th^ and 75^th^ percentiles, and the lines extending from the box are 1.5 interquartile ranges from the median. Data points outside of the 1.5 interquartile ranges are represented as dots.

In the active layer, dissolved organic nitrogen (DON = TDN − DIN) dominated the TDN pool representing ~78% of TDN in both mineral and organic layers. The DIN contribution increased with depth and ammonium-N dominated the DIN pool, with its concentration reaching 0.410 ± 0.326 mg N-NH_4_^+^ g^−1^ soil at 1–2 m depth in organic permafrost layers (Supplementary Fig. 3). In contrast, nitrate concentrations remained very low in both mineral and organic soils with 67 of 220 samples having NO_3_^−^ concentrations below our instrument detection limit (0.006 mg N L^−1^, Fig. 2, Supplementary Fig. 3). The TDN, NH_4_^+^, and NO_3_^−^concentrations measured in this study were within the range of values reported for permafrost soils^6,27–29^.

While the active layer and mineral permafrost layers contained relatively little DIN, the pool of NH_4_^+^ in the organic permafrost layers (32.8 [11.3–66.2] g N–NH_4_^+^ m^−3^, Fig. 2) was very large compared to the general low nitrogen availability in Arctic environments^13^. Similar enrichment in NH_4_^+^ were reported in various permafrost settings in Sweden, Siberia, and Greenland^28–30^ as being a particular feature of permafrost soils in the Arctic. Saturated and oxygen-limited conditions in peatlands and syngenetic permafrost aggradation likely prevented microbial processing and/or leaching of NH_4_^+^ out of the soil profile. Seasonal NH_4_^+^ leaching down through the active layer at the end of the growing season, leading to NH_4_^+^ accumulation at the permafrost table during the winter freeze-up, likely contributed to the accumulation of a large pool of NH_4_^+^ ^28^ in the solute-enriched permafrost transition zone^31^ on decadal to centennial time scales. In addition, as microorganisms in permafrost soils are active under freezing conditions^32^, and some pore water remains within the permafrost, at least some of the large NH_4_^+^ pool likely originates from in-situ mineralization below 0 °C over thousands of years^6,28^. A thaw-induced release of permafrost NH_4_^+^ of this magnitude will likely affect Arctic terrestrial and aquatic ecosystems changing trophic structures and providing new habitat niches^13^ while altering landscape-wide carbon cycling processes^15^.

### Optical properties of DOM in permafrost soils

**Fig. 3:**
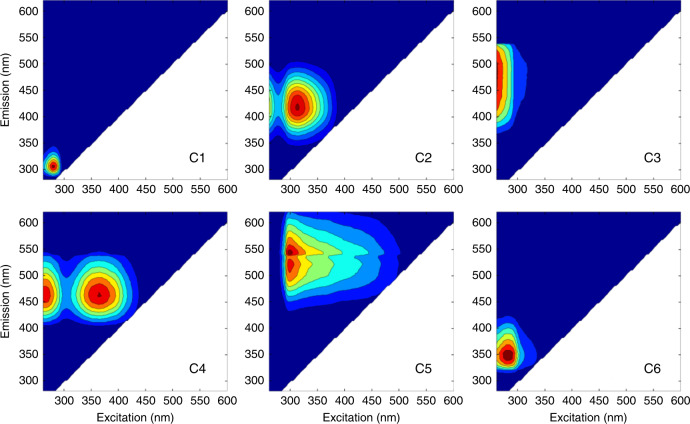
Six fluorescent components identified using PARAFAC analysis. Excitation and emission peak positions of the three-dimensional excitation-emission matrices of the independent components are indicated alongside descriptions in Table 1.

The PARAFAC modeling for DOM fluorescence was conducted in all permafrost soils, spanning a wide range of surficial geology and climate conditions. We related six identified fluorescent components to fractions previously reported in surface waters in northern latitudes (Fig. 3, Table 1). Components C2, C3, C4, and C5 were previously related to humic-like or fulvic-like components associated with high molecular weight (HMW), aromatic organic compounds originating from terrestrial sources such as plant inputs and SOM^33–35^. Components C1 and C6 are ubiquitous across a wide range of terrestrial and marine environments and correspond, respectively, to tyrosine-like (C1) and tryptophan-like (C6) components, representing proteinaceous compounds from microbial activity such as amino acids, peptide materials and free or bound proteins^34^.

**Table 1:** Description of the six components identified by PARAFAC of 226 excitation-emission matrices.

Component	Excitation max (nm)	Emission max (nm)	Description and likely structure	Cory and McKnight (2005)	Fellman et al. (2010)	Mann et al. (2016)	Walker et al. (2013)	Openfluor comparison^a^	Source of DOM
				Polar lakes and other freshwaters	Review	in main Arctic rivers	in main Arctic rivers	(component with TCC > 0.95)	
1	275	306	Tyrosine-like—Amino acid, soluble bound microbial DOM, may indicate more degraded peptide material	Comp 13	C1	-	-	15	Terrestrial, Autochtonous, Marine
2	310	415	UVC humic-like—High molecular weight, syringaldehyd-like (byproducts of lignin breakdown), associated with high DOM contents	Comp 10	C7	AG3	C1	23	Terrestrial
3	<255	450–500	UVA humic-like or fulvic like—High molecular weight, syringaldehyd-like (byproducts of lignin breakdown), associated with high DOM contents	Q2 (Comp 2)	C5	AG1	C4	12	Terrestrial
4	360 (<255)	460	UVC humic-like—High molecular weight and aromatic humic acid	SQ2 (Comp 7)	C10	AG4	C2	22	Terrestrial
5	295–300	520–540	Semiquinone-like—High molecular weight and highly aromatic	SQ1 (Comp 5)	–	–	C3	1^b^	Terrestrial
6	280	345	Tryptophan-like—Amino acid, soluble free or bound microbial DOM, may indicate intact proteins or less degraded peptide matieral	Comp 8	C2	AG7	C5	24	Terrestrial, Autochtonous, Marine

^a^number of studies in the open fluorescence database which previously identified components displaying similar optical properties (tucker congruence coefficient, TCC > 0.95).

^b^Excitation has a TCC = 0.95.

**Fig. 4:**
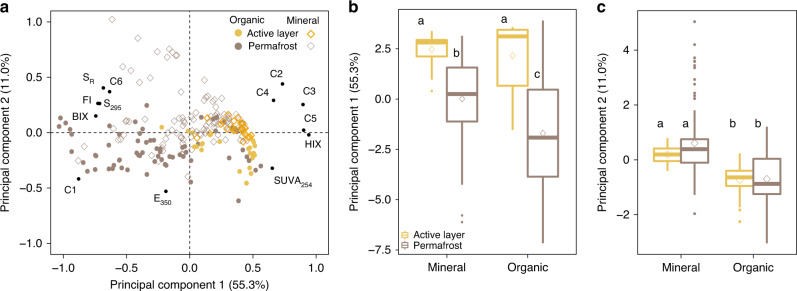
Principal component analysis of DOM optical properties. The analyses takes into account the relative contribution of PARAFAC components and optical indices: the absorption coefficient at 350 (E_350_), the specific UV absorbance (SUVA_254_, L mg C^−1^ m^−1^), the absorption spectral slope over the spectral band 275–295 nm (S_295_), the slope ratio between S_295_ and the spectral slope over 350–400 nm (S_R_), the fluorescence index (FI), the freshness index (BIX), the humification index (HIX). **a** Explanatory variable loadings are shown as black dots and scores across the first and second principal components for all samples labeled by layer type—organic layers are shown as circles and mineral as diamonds—and colored by location in the active layer or permafrost. **b** Box plot of the scores of the first principal component for organic (soil C ≥ 12%) and mineral layers (soil C < 12%) in the active layer and in the permafrost. **c** Box plot of the scores of the second principal component for organic (soil C ≥ 12%) and mineral layers (soil C < 12%) in the active layer and in the permafrost. In each box plot, the diamond represents the mean, the horizontal line represents the median, the end of the box the 25^th^ and 75^th^ percentiles, and the lines extending from the box are 1.5 interquartile ranges from the median. Data points outside of the 1.5 interquartile ranges are represented as dots. Letters indicate significant differences between thermal layers and soil types indicated by Mann–Whitney U test.

The optical properties of DOM differed between thermal layers and between organic and mineral soil leachates (Fig. 4). Permafrost DOM exhibited a distinctive signature with a greater proportion of low molecular weight (LMW) proteinaceous organic compounds than found in the active layer (Fig. 4). In the active layer, the HMW aromatic fluorophores derived from terrestrial sources were the most abundant in both mineral and organic layers (94.7% [88.3–96.7] of the total fluorescence). These fluorophores contributed less to the fluorescent DOM, representing 77.0% [57.9–88.6] and 40.7% [23.9–70.8] of the fluorescence in permafrost mineral and organic layers, respectively (Supplementary Fig. 4). The tyrosine-like component (C1) was the most abundant fluorophore in the organic permafrost layers accounting for 51.2% [22.4–62.9] of the total fluorescence, and the tryptophan-like component (C6) representing 7.2% [5.0–10.1]. In the mineral permafrost, the tyrosine-like and tryptophan-like components represented 12.8% [2.7–34.4] and 7.6% [5.5–11.0] of the total fluorescence, respectively (Supplementary Fig. 4). These components contributed less in the active layer than in the permafrost representing 5.3% [4.1–22.8] in organic and 5.3% [3.3–10.7] in mineral layers of the fluorescent DOM (Supplementary Fig. 4).

The multivariate statistical analyses based on absorbance and fluorescence indices demonstrated that permafrost DOM exhibited a distinct optical signature shared by both mineral and organic layers (Fig. 4a, b). The permafrost leachates were enriched in LMW proteinaceous compounds with low aromaticity while the active layer DOM was comprised of HMW aromatic condensed organic compounds. The fluorescence index FI (a common proxy for the relative contribution between microbial and terrestrial DOM) and the ratio of absorption spectral slopes S_R_, which decreases with molecular weight and aromaticity, increased with depth (Fig. 4a, b, Supplementary Fig. 5) indicating more protein-derived substrate with a lower average molecular weight in the permafrost^34^. HIX and SUVA_254_ values, which are known to increase with bulk aromaticity and condensing, decreased with depth and in the permafrost (Supplementary Fig. 5) suggesting that permafrost stores less condensed and aromatic DOM than in the active layer^20,34^. Similar values of HIX and SUVA_254_ in active layer mineral and organic layers illustrate the similar long-term microbial processing of organic matter in the seasonally thawed zone Supplementary Fig. 5). In both thermal layers, DOM in organic and mineral layers only differed by the absorption at 350 nm, with organic layers displaying higher absorption values due to greater concentrations of aromatic DOM^33^ (Fig. 4a, c).

Our values of optical indices are consistent with the few available characterizations of DOM in the active layer and permafrost^5–8,25,36^. Due to different organic matter sources, DOM in mineral permafrost layers has been previously characterized by lower SUVA_254_ values ([0.6–1.2] L mg C^−1^ m^−1^)^5,8^ than organic active layer materials ([1–4.5] L mg C^−1^ m^−1^)^6,8,25,37^ due to a relative enrichment in microbial exometabolites and root exudates^37^ in mineral soils compared to peaty organic layers. Last, molecular techniques also support our findings by showing enrichment of LMW compounds such as carbohydrates^8^, acetate and butyrate^5^ in the permafrost in Alaska and Siberia.

### Controls of the pan-Canadian permafrost DOM pool

Even though permafrost DOM displays contrasting optical properties from active layer DOM, the signature of permafrost DOM is weakly detected within Arctic catchments. This is mainly due to the high reactivity of DOM, which experiences sorption and desorption when percolating in the soil^25^, photo-oxidation of HMW aromatic components in streams and rivers, and microbial processing of less aromatic compounds^10,18,25^. Indeed, while our results show the high contribution of tyrosine-like component to the fluorescent DOM in both mineral and organic permafrost in the Canadian Arctic region, studies that have conducted PARAFAC modeling on DOM in Arctic surface waters have only detected a low contribution of this component in headwater catchments^38,39^, with no contribution at all in Arctic rivers^33,35^. The major tyrosine contribution to the permafrost fluorescent DOM and its disappearance within the fluvial continuum provides additional evidence of its high reactivity^5,10,19^.

Following permafrost thaw, three main processes lead to the decrease in the proteinaceous contribution during lateral flow downslope along the permafrost table, prior to reaching water bodies. First, soil microbes could preferentially mineralize tyrosine-like DOM components, such as amino acids and oligopeptides^40^, as they utilize aliphatic compounds^41^ almost immediately following permafrost thaw^5^. Recent studies in Arctic permafrost environments highlight that low SUVA_254_^19^, high S_R_^42^ values and a high contribution of tyrosine-like^43^ correlated with greater lability of bulk DOM^6,36,43,44^. Therefore, we suggest that Canadian permafrost likely contains a high proportion of biodegradable DOM. Secondly, in northern ecosystems plants take up amino acids and oligopeptides directly as a source of N, leading to a decrease in proteinaceous compounds in exported waters^45^. Finally, the retention of less aromatic compounds and protein-like fluorophores in mineral subsoils and the preferential release of HMW aromatic fluorophores lead to the depletion of tyrosine-like components in riverine fluorescent DOM and the relative enrichment in aromatic compounds^25^.

**Fig. 5:**
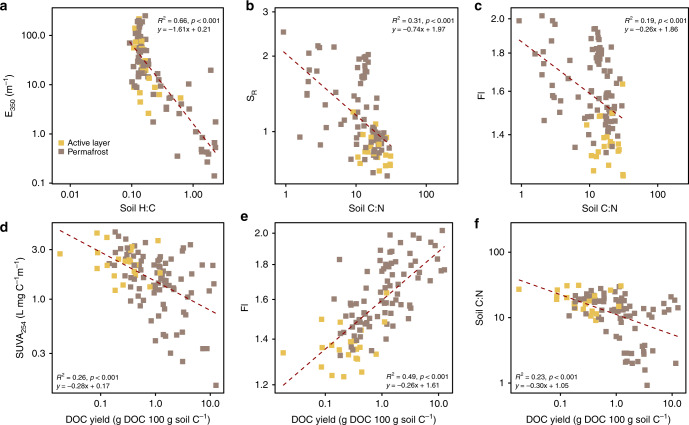
Relationships between indices of DOM character and soil properties. Relationships between E_350_ (**a**), S_R_ (**b**), FI (**c**, **e**), SUVA_254_ (L mg C^−1^ m^−1^) (**e**)) and soil properties (H:C (**a**), C:N (**b**, **c**, **f**), and DOC yield (g DOC 100 g soil C^−1^) (**d**, **e**, **f**)) for both active layer and permafrost samples. Each point represents one sample. The linear regression lines and equations are for active layer and permafrost samples.

Our results demonstrate robust relationships between SOM stoichiometry (C:N and H:C ratios) and chromophoric and fluorescent DOM properties and DOC yield highlighting the control of permafrost organic matter on the DOM pool (Fig. 5). E_350_ decreased with increasing H:C ratios (Fig. 5a), suggesting that chromophoric DOM abundance was greater for C-rich SOM^24^. The C:N ratio was negatively correlated with S_R_ and FI (Fig. 5b, c) demonstrating that N-rich SOM (i.e., enriched in microbial biomass) yields LMW DOM with low aromaticity. In addition, SOM properties (i.e., C:N ratio), the degree of aromaticity (i.e., SUVA_254_) and the microbial origin of fluorescent DOM (i.e., FI) was correlated with the water extractable DOC yield (Fig. 5d–f). Therefore, permafrost samples with lower C:N ratio yield proportionally more DOM that contains a higher proportion of less aromatic and more proteinaceous compounds, which are likely labile^44^ relative to the active layer. As soil C:N and H:C ratios are widely measured in permafrost soils^23^, these findings allow for upscaling of site or plot level analyses to a circumpolar characterization of the DOM pool.

Our results further indicate that the uppermost permafrost degradation has the potential to release a larger amount of DOC and TDN (mostly as DON and NH_4_^+^) than the active layer currently releases to high-latitude terrestrial and aquatic ecosystems. These findings are in agreement with observations of thawing permafrost leading to shifts in the supply of carbon and nutrients to surface waters^46,47^. Using the NCSCDv2 dataset^3^ for the pan-Canadian permafrost area, we estimated the DOC pool available right upon thaw increasing from 0.2 [0.2–0.5] Pg C in 0–1 m, to 1.1 [0.7–2.1] Pg C in 1–2 m and decreasing to 0.9 [0.7–1.5] Pg C in 2–3 m depth interval. Although DOC is a relatively small component of the total permafrost carbon pool, its depletion through hydrological mobilization will likely contribute relatively fast to soil carbon loss in Arctic landscapes^48^.

**Fig. 6:**
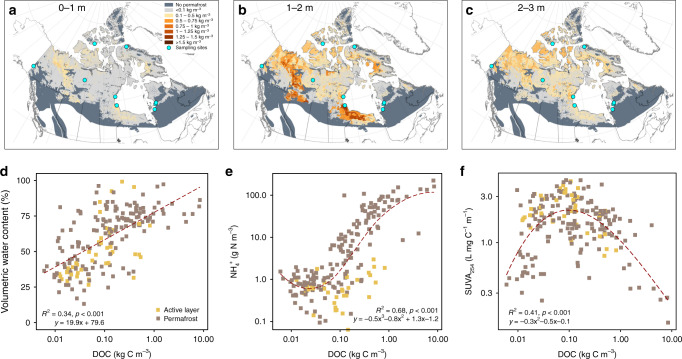
Maps and controls of the DOC pool in the pan-Canadian permafrost area. **a**–**c** Maps of pool of DOC (kg C m^−3^) that would be available upon permafrost thaw for three soil layers: 0–1 m, 1–2 m, 2–3 m. The DOC pool in permafrost soils was estimated by multiplying soil carbon pool of the three different gelisol types from the NCSCDv2 by the median of water extractable DOC yield of mineral and organic soils. Blue dots represent the locations of the coring sites. The dark gray area represents the non-permafrost soils within the Canadian permafrost distribution zone (isolated, sporadic, discontinuous and continuous). **d** Volumetric water content (%), **e** pool of NH_4_^+^ (g N m^−3^), and **f** SUVA_254_ (L mg C^−1^ m^−1^) as a function of the pool of DOC (kg C m^−3^) for both active layer and permafrost samples. Each point represents one sample. The regression lines (dashed lines) and equations are for active layer and permafrost samples combined.

In addition to an increase in DOC availability in the large areas of peatlands located in southern boundaries of the permafrost region (i.e., the Mackenzie River region, the Hudson Bay lowlands), which have been subject to rapid thaw^49^ (Fig. 6a–c), the DOC pool increased with volumetric water content (Fig. 6d). While being more prone to thermokarst processes^2^, the degradation of ice-rich permafrost could potentially export more carbon to surface waters. Our data further show that the DOC pool was positively correlated with the NH_4_^+^ pool and the large DOM pools are characterized by lower aromaticity (i.e., low SUVA_254_) (Fig. 6e, f). This finding supports reported observations of the accumulation of NH_4_^+^ and biodegradable DOC in Arctic permafrost soils^47^. While proteins compose most of the DON that are thereafter degraded to amino acids and mineralized to NH_4_^+^, we demonstrate here the buildup of both NH_4_^+^ and tyrosine-like fluorophores in the permafrost, which then halted their decomposition and export. As reported in glacier ice, the dominant contribution of the tyrosine-like component to fluorescent DOM is evidence of microbial metabolism in permafrost^50^. The distribution of ground ice has been proven to be the main factor controlling the fate of permafrost carbon (i.e., thermokarst activity and methane production)^2^. Our data demonstrate that ice-rich permafrost soils have the potential to release great amount of NH_4_^+^ and DOM characterized by a specific low aromaticity protein-rich optical signature that suggests rapid mineralization potential.

The uppermost layers of Canadian permafrost, found immediately below the seasonally thawing active layer, currently store substantial DOC and NH_4_^+^ pools, particularly in organic permafrost soils. This suggests that permafrost thaw—in addition to the release of reactive DOM—is likely to enhance nutrient inputs and availability into terrestrial, as well as aquatic systems. This nutrient priming mechanism will almost certainly interact with and affect microbial processing of organic matter and primary productivity, altering the net carbon balance of Arctic ecosystems^15^. Here, we show that permafrost DOM exhibits a highly consistent and distinctive chemical composition across a very large region of northern Canada and spanning numerous distinct environments and climates. In conclusion, our results support the premise that degradation of organic rich permafrost will increase carbon release and potentially affect aquatic systems through carbon and nutrient additions.

## Methods

This study initiated from work conducted as part of the Natural Sciences and Engineering Research Council of Canada (NSERC) Discovery Frontiers project Arctic Development and Adaptation to Permafrost in Transition (ADAPT), which brought together 15 laboratories from across Canada to develop an integrated Earth systems science framework on diverse aspects related to thawing permafrost conditions in the Canadian Arctic^22^. Data produced with the standard protocols for cryostratigraphy and soil analyses from the ADAPT project are published through the Nordicana D database^51^ and briefly described below.

### Permafrost cores

We investigated 25 soil cores that were collected by members of the ADAPT project teams^22^ in nine distinct regions across the Canadian Arctic (Table 1). The cored areas represent a broad range of Canadian permafrost conditions, soil types, and landscapes covered by diverse ecosystems such as boreal forest, palsa peatlands, and various tundra vegetation, including dry heath, mesic shrub tundra, and wet sedge. Each soil core was obtained in 2012 and 2013 and consisted of the seasonally thawing active layer and uppermost permafrost soil layers. Five sites lack active layer samples (Arviat 1A, SAS A and B, VDT A and B). After retrieval, the soil cores were kept frozen in the field and shipped frozen to Laval University, Quebec City, Quebec, Canada. Here, the soil cores were stored frozen until subsampled into 5 cm segments in 2014. In the following analyses, each meter of a soil core contributes 3–10 subsamples. In each sampled region, we obtained duplicate soil cores from at least two distinct sites differing in soil and/or vegetation type, soil moisture, and topography. Measurements of soil moisture and bulk density and the water extracts were performed at Queen’s University on frozen soil core samples. All soil samples were classified as located in either active layer or permafrost soil layers according to stratigraphic information based on CT scans and site descriptions and classified as organic or mineral layers following the USDA Soil Taxonomy^52^. Our active layer thickness, i.e., maximum thaw depth during summer, is indicative of sampling year and it does not take decadal and local spatial variability into account.

### Soil properties

After being oven dried at 105 °C, all subsamples were analyzed for total mass content of carbon (C), nitrogen (N), and hydrogen (H) by combustion using a LECO CHN628 Elemental Analyzer (LECO Corporation, St. Joseph, MI) at the Centre d’Etudes Nordiques (CEN) Radiocarbon Dating Laboratory at Laval University, Quebec^51^. At Laval University, prior to combustion, we determined bulk density and gravimetric water content and homogenized the subsample, using a mortar and pestle. For additional subsamples, conventional ^14^C dating was done at the CEN Radiocarbon Laboratory, and samples for ^14^C-AMS dating were prepared at CEN and dated at the University of California Riverside radiocarbon facility (selected sites only; Table S1). Further information about CHN and radiocarbon analyses are available http://www.cen.ulaval.ca/en/page.php?lien=labradio.

The total carbon content comprises organic and inorganic carbon, soil samples were not acidified prior to analyses. Based on site descriptions, peat properties (i.e., pH, parental material), no studied soils were developed from carbonate bedrock, and soil C and N contents were strongly correlated in mineral soils (*r* = 0.98, *P* < 0.001), and previous studies in the same sites^22,53–55^, we assume very little inorganic carbon (~<1%) contribute to the total carbon content in both organic and mineral layers.

Due to the lack of clay-rich layers with more than 2% C, we consider soil materials as mineral layers when the C content was below 12% (by weight) and as organic layers when C content was above 12% C (by weight)^52^.

### Water extracts and analyses

For all active layer and permafrost soil samples, 20 g of frozen soil from a 5 cm segment was transferred to a glass beaker and allowed to thaw at room temperature for 30 min before 100 mL of deionized water was added (mass to volume ratio 1:5). Each soil and water slurry was homogenized for 1 h, using a shaker table, after which slurries were allowed to settle for 30 min before vacuum-filtration through glass fiber filters (Fisher G4; 1.2 mm pore-size). Water extracts were stored frozen in HDPE vials until further analyses. We analyzed water extracts of these soils for dissolved organic carbon (DOC) and nitrogen (DTN), ammonium (NH_4_^+^) and nitrate (NO_3_^−^), and we characterized the composition of DOM using UV-Vis absorbance and fluorescence spectroscopy. All analyses were conducted in the Department of Geography and Planning at Queen’s University. DOC and TDN measurements were performed simultaneously using high-temperature combustion on a Shimadzu TOC-V Analyzer with a TMN-1 chemiluminescence detection unit (Shimadzu North America, Columbia, MD). Milli-Q blanks were run at the beginning and throughout every run to ensure consistency. A new calibration was generated for each DOC and TDN run and based on replicate analyses of standards, which were analyzed within every run. DOC and TDN were calculated as the mean of between three and five injections with the coefficient of variance always <2%. For DOC analyses, the detection limit was 0.14 mg L^−1^ and analytical errors were 2.9%. For TDN analyses, the detection limit was 0.02 mg L^−1^ and analytical errors were 4.0%. Concentrations of NH_4_^+^ and NO_2_^−^-NO_3_^−^ were measured by colorimetry using a flow Analyzer (Astoria-Pacific 2-channel Flow Analyzer, EPA Method 353.2). For NH_4_^+^, the detection limit was 0.006 ppm N and the analytical error was 2.6%. For NO_3_^−^, the detection limit was 0.006 ppm N and the analytical error was 1.6%. Method Detection limit was determined by running a low-concentration standard when samples were run, and calculated as concentration of DOC/TDN/NH_4_^+^/NO_3_^−^ in the blanks over all the runs plus thrice standard deviation of the concentrations of DOC/TDN/NH_4_^+^/NO_3_^−^ in the low-concentration standard over all the runs. The analytical error was calculated as the ratio of the standard deviation of the concentrations of DOC/TDN/NH_4_^+^/NO_3_^−^ in the ‘method check’ standard multiple times over all runs by the average concentrations of DOC/TDN/NH_4_^+^/NO_3_^−^ in the ‘method check’ standard multiplied by 100. All calculations of water extracts sample elemental contents and pools were corrected for dilution associated with the deionized water addition and sample-specific gravimetric soil moisture content.

We applied seven different indices to investigate the DOM optical properties: the absorption coefficient at 350 (E_350_), the specific UV absorbance (SUVA_254_, L mg C^−1^ m^−1^), the absorption spectral slope over the spectral band 275–295 nm (S_295_), the slope ratio between S_295_ and the spectral slope over 350–400 nm (S_R_), the fluorescence index (FI), the freshness index (BIX), the humification index (HIX). Excitation-emission matrices (EEMs) were collected and decomposed into individual fluorescent components using a parallel factor analysis (PARAFAC).

UV-visible absorbance and fluorescence measurements and Emission Excitation Matrices were collected at room temperature using a Horiba Aqualog (Horiba-Jobin Yvone Scientific Edison, NJ). Each sample was run individually in 1-cm path length quartz cuvette. Absorbance spectra were blank-corrected using Milli-Q water. UV-visible absorbance spectra and excitation values were collected between 240 and 600 nm at 3 nm intervals and emission wavelengths were scanned from 214.16 to 621.03 nm at 3.15 nm intervals. The samples were run at varied integration time and gain settings to optimize the quality of the results for each sample. EEMs files were corrected for blanks and inner filter effects, 1st and 2nd order Rayleigh scatter effects were removed using the manufacturer correction procedure. The signal was normalized using a 1 ppm Quinone Sulfate standard, made up in 0.05 M H_2_SO_4_, and run daily during measurements, to account for fluctuations in the instrument light source characteristics. All intensities were reported in terms of Quinone Sulfate Units (QSU).

Absorbance data were converted to Napieran absorption coefficients (E in m^−1^) by multiplying raw absorbance values by 2.303 and dividing by the cuvette path length (m)^56^. E_350_ was calculated and represents a quantitative measure of the chromophoric fraction of DOM that is positively correlated with DOC and lignin phenol content^57^. SUVA_254_ was calculated as the decadal UV absorbance at 254 nm divided by the DOC concentration^20^. SUVA_254_ is positively correlated to bulk DOM aromaticity because aromatic compounds absorb more light in the UV-visible spectra. The DOM derived from plant and soil organic matter dissolution, which is composed of large biopolymers, shows higher aromaticity than autochthonous and microbially derived DOM that comprised small biopolymers and monomers assimilable and produced by microorganisms^20,58^. The absorption spectral slope S_295_ was calculated over the spectral band 275–295 nm. S_R_ was calculated as the ratio between S_295_ and the absorption spectral slope over 350–400 nm^59^. S_295_ and S_R_ differ between DOM sources being indicators of molecular weight and aromaticity. Higher slope coefficients are associated with lower molecular weight and decreasing DOM aromaticity^18,59,60^.

FI, which was calculated as the ratio of emission at 470 to 520 m at an excitation wavelength of 370 nm^61,62^, illustrates the source of DOM from terrestrially derived (plant and soil organic matter, ~1.2) to microbial (bacteria and algae by-products, ~1.8)^34^. BIX is calculated as the ratio of emission at 380 nm divided by the emission intensity maximum observed between 420 and 435 nm at an excitation wavelength of 310 nm^63^. BIX is an indicator of the relative freshness of the bulk DOM, increasing with more recently derived DOM^34^. HIX was calculated as the area under the emission spectra 435–480 nm divided by the peak area over two spectral bands 300–345 nm + 435–480 nm, at an excitation of 254 nm^34,64^. An increase in HIX is associated with a shift of emission spectra toward longer wavelengths and illustrates the increased contribution of HMW aromatic fluorescing molecules with lower H:C ratios^34^.

PARAFAC models decompose data that are arranged in three-dimensional arrays (sample × excitation wavelength × emission wavelength) into accurate spectra and relative concentrations of known fluorescent groups showing similar chemical composition in a complex DOM mixture^21,65^. PARAFAC individual components refer to groups as ‘humic-like, fluvic-like or protein-like’ with various properties rather than pure organic molecules^34^.

PARAFAC modeling was performed using the DOMFluor toolbox within MATLAB^66^ in order to decompose EEMs into independent fluorescent components. Excitation wavelengths below 255 nm and emission wavelengths below 280.81 were removed from matrices in order to avoid deteriorating signal-to-noise ratios^67^. Prior to modeling, regions of the spectra influenced by 1st and 2nd order scatter peaks and with no fluorescence (i.e., emission << excitation) were cut and replaced by missing values. A six-component model was generated incorporating a total of 226 samples. Model parameters were constrained to be non-negative. The model, which identified components with unimodal emission maxima, was validated using residual analysis characterized by instrument noise and a lack of systematic pattern, split-half analysis and random initialization^21,66^ and explained 99.9% of total the fluorescence signature.

PARAFAC component intensities were normalized to the sum of component fluorescence intensities and expressed as their relative contribution to the total fluorescence. The identified component emission and excitation loadings were compared with the open fluorescence database^68^ (https://openfluor.lablicate.com/) and with previous studies using PARAFAC analysis that are not included in the database. Components have been previously reported by 97 independent studies from openfluor database in various environments (Tucker Congruence Coefficient, TCC > 0.95) and in many studies in Arctic ecosystems (Table S4).

### Statistical analyses

All statistical analysis were carried out using the R free software version 3.2.1^69^. Bartlett’s and Shapiro-Wilk tests were performed to, respectively, evaluate the variance homogeneity and the normal distribution of each variable of interest. Even log-transformed most variables were not normally distributed. A Principal Component Analysis (PCA) was performed using PARAFAC components and absorbance and fluorescence indices (biogeochemical data and soil properties were not included) was used to examine the differences in optical properties between thermal layers and layer natures. Non-parametric analyses of variance using Kruskal-Wallis (defined by its *chi-squared H and P value)* and the multiple pairwise-comparisons using pairwise Wilcoxon rank sum test (defined by its *p value*) were used to determine significant differences in concentrations and yields of DOC, DTN, NH_4_^+^, NO_3_^−^ and optical properties among thermal layers (active layer vs. permafrost), and between soil layer types (organic vs. mineral). Wilcoxon rank sum test tests (defined by *W* and *P value*) were used to assess the significance of difference between two specific groups. Before performing the PCA auto scaling was conducted on all variables with the FactoMineR package in order to decrease the leverage of high values. Regression analyses were employed to determine relationships between concentrations and yields of DOC, DTN, NH_4_^+^, NO_3_^−^, optical properties and soil properties.

### Canadian Permafrost landscape DOC pool estimates

We estimated the DOC pool in permafrost soils at the pan-Canadian scale using the updated version of the Northern Circumpolar Soil Carbon Database (NCSCDv2)^70^ at a spatial resolution of 1 km. NCSCDv2 provides information on SOC storage stratified both by depth layers and soil types. In the database, the class of Gelisol (permafrost soils) is subdivided in 3 suborders: Histel, Turbel, Orthel. We classified the soil type of each borehole as “organic permafrost soils” (Histels) or “mineral permafrost soils” (Orthels and Turbels). Soils were defined as “organic permafrost soils” if the organic surface layer was thicker than 40 cm. We defined soils as “mineral permafrost soils” for both Turbels (cryoturbated soils) and Orthels (non-cryoturbated), if the organic layer was less than 40 cm thick^52^. Non-permafrost soils were not taken into account in any calculations.

To create maps of soil DOC, we multiplied maps of soil carbon by the median yield of water-extractable DOC per soil type and at three depth layers (0–1 m, 1–2 m, 2–3 m). We did not calculate different yields between the active layer and permafrost layers. We examined the changes in yield between soils and depth layers using Kruskal–Wallis and Mann–Whitney test. We used quadrature to combine uncertainty of DOC yields with the 14.5% uncertainty in soil carbon from the NCSCD. We used the median and the 25^th^ to 75^th^ percentiles as the representative DOC yield and range of uncertainties, respectively.

## Supplementary information

Supplementary Information

Supplementary Dataset 1

Supplementary Dataset 2

Supplementary Dataset 3

## Data Availability

The authors declare that all data supporting the findings of this study other than the excitation-emission matrices (EEMs) and the PARAFAC parameters are available within the paper and its supplementary information and supplementary data files. The EEMs and the PARAFAC code that generated the 6-component model are available from the corresponding author upon request. All data associated with this study will shortly be posted on the Polar Data Catalogue metadata website (https://www.polardata.ca) and Nordicana-D, the online data report series archived by the Centre for Northern Studies (http://www.cen.ulaval.ca/nordicanad/en_index.aspx).
