# Supplementary material for: Canadian permafrost stores large pools of ammonium and optically distinct dissolved organic matter
Source: Nat Commun. 2020 Sep 9;11:4500. doi: 10.1038/s41467-020-18331-w (PMC7481224; doi:10.1038/s41467-020-18331-w)
Supplement: Supplementary file 1 — Supplementary Information [file 41467_2020_18331_MOESM1_ESM.pdf]

Supplementary information for

Canadian permafrost stores large pools of ammonium and optically distinct dissolved organic matter

by Fouché et al.

Supplementary Figures

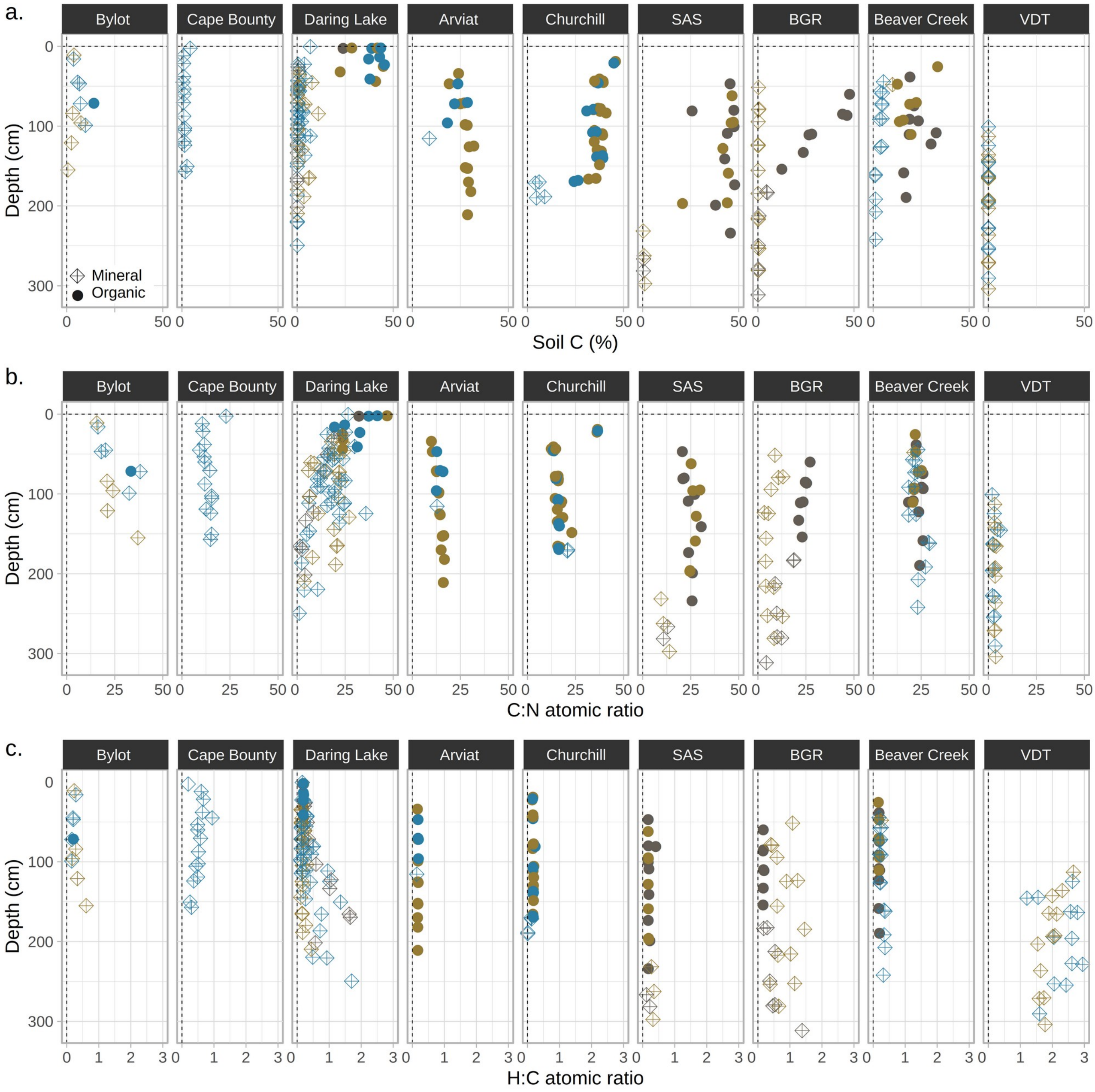

Supplementary Figure 1. Profiles of a) soil carbon content, b) atomic C:N ratios and c) atomic H:C atomic ratios for the nine study sites. Values on both axes are fixed. Organic layers (soil C ≥ 12%) are shown as circles and mineral layers (soil C < 12%) as diamonds. Colours represent the different boreholes at each site.

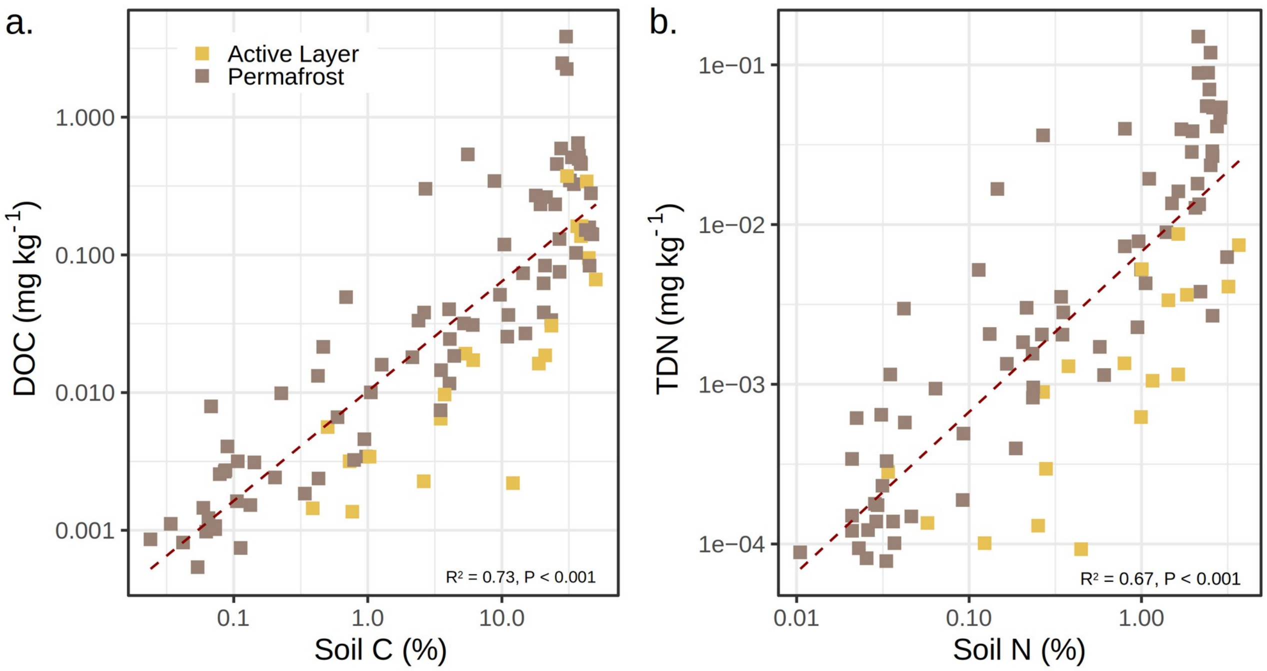

Supplementary Figure 2. Relationships between a) DOC concentration and soil carbon (C) content and b) TDN concentration and soil total nitrogen (N) content for both active layer and permafrost samples. Each point represents one sample. The regression lines and equations are for all active layer and permafrost samples.

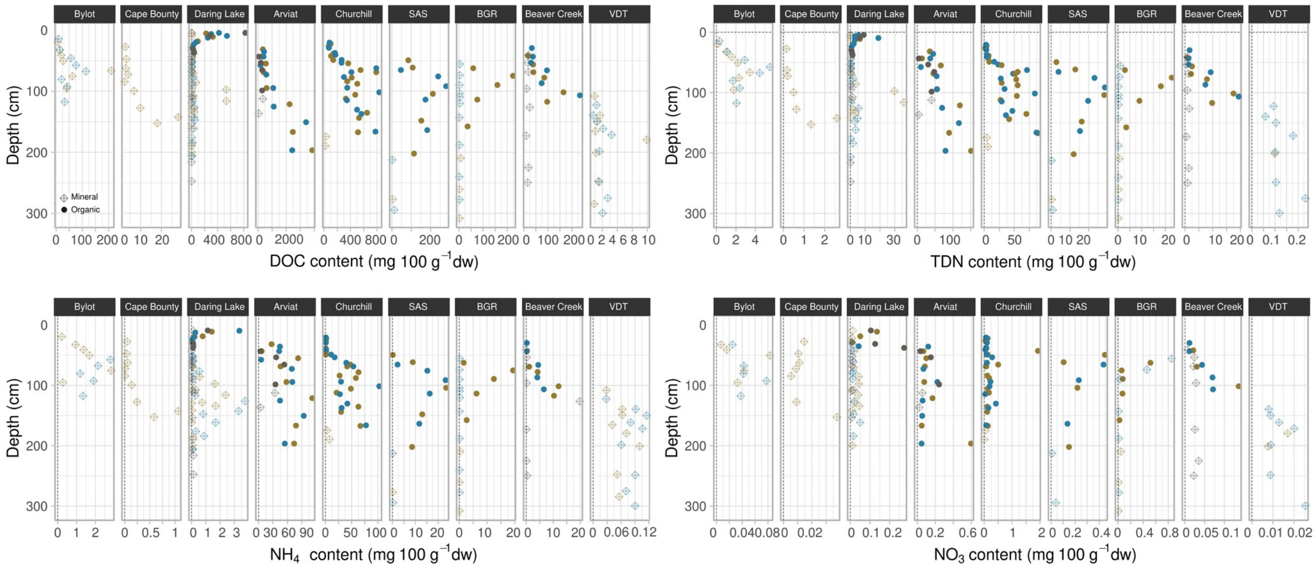

Supplementary Figure 3. Profiles of contents of a) dissolved organic carbon (DOC), b) dissolved total nitrogen (TDN), c) ammonium (NH_4^+) and d) nitrates (NO_3^-) for the nine study sites. Axis values vary between sites. Organic layers are shown as circles and mineral layers as diamonds. Colours represent the different boreholes at each site.

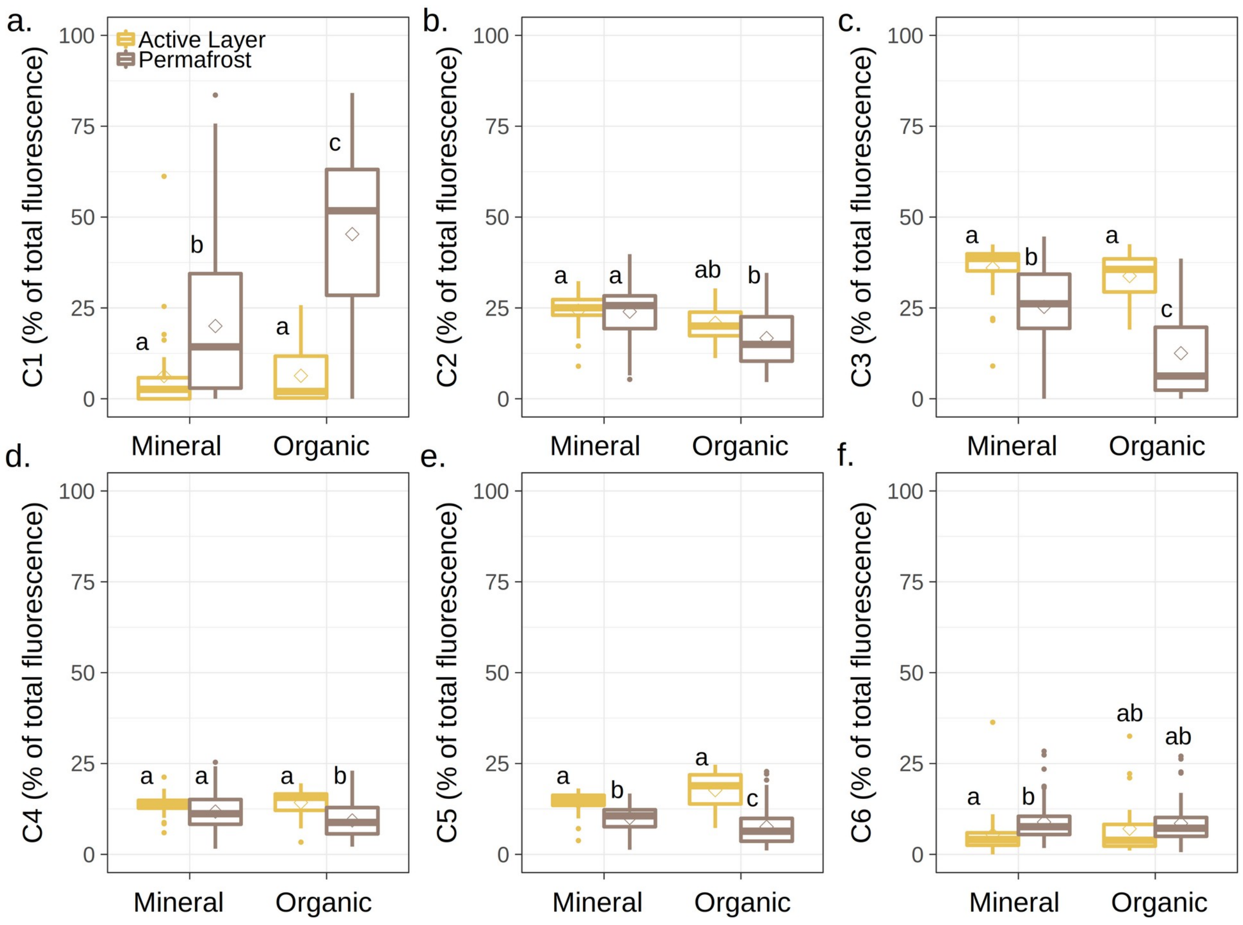

Supplementary Figure 4. Relative contribution of the six components decomposed using PARAFAC modelling (Figure 2) in the active layer and in the permafrost grouped by layer type with the following classification: organic layers (soil C ≥ 12%) and mineral layers (soil C < 12%). Letters indicate significant differences between thermal layers and soil types indicated by Mann-Whitney U test. The box plots summarize the distribution of component contributions for each layer type and in the active layer and permafrost. Boxplots represent 25–75% quartiles and whiskers are 1.5 interquartile ranges from the median. Medians and means are shown as lines and diamonds, respectively.

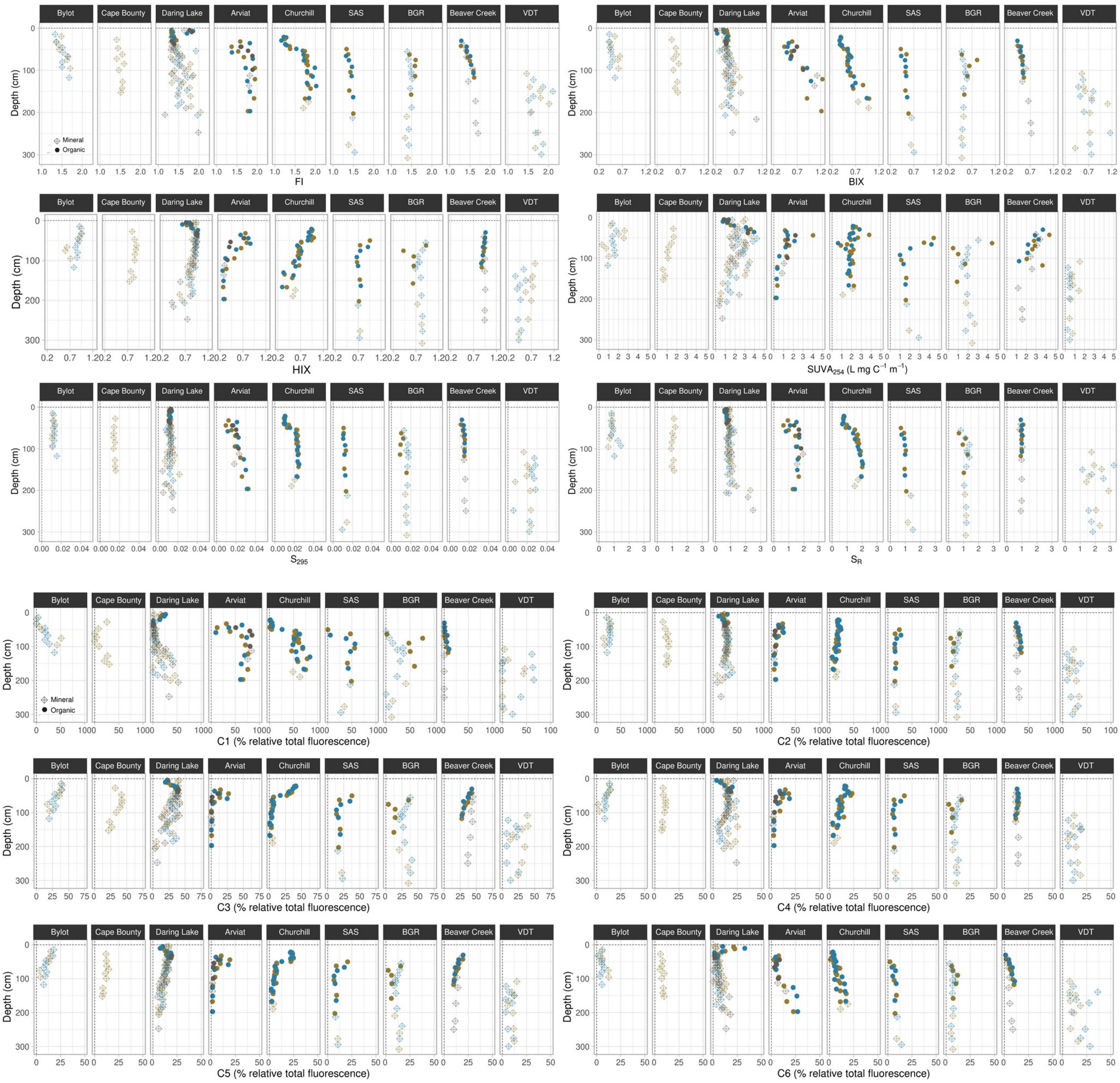

Supplementary Figure 5. Profiles of the optical indices and relative contributions of the six PARAFAC components (% of the total fluorescence) for the nine study sites. Profiles of a) the fluorescence index (FI), b) the freshness index (BIX), c) the humification index (HIX), d) the specific UV absorbance ($SUVA_{254}$, L mg C^{-1} m^{-1}), e) the absorption spectra slope over the spectral band 275-295 nm (S_{295}) and f) the slope ratio between S_{295} and the spectra slope over 350-400 nm (S_R). For optical indices axis values are not fixed while for PARAFAC components are fixed for all sites. Organic layers are shown as circles and mineral layers as diamonds. Colours represent the different boreholes at each site.
